# Inclusion of Children with Disabilities in Disaster Management

**DOI:** 10.3390/children8070581

**Published:** 2021-07-07

**Authors:** Jeong-Hun Jang, Kyoo-Man Ha

**Affiliations:** 1 YB Ophthalmic Clinic, Suwon 16490, Korea; Slit@daum.net; 2 Department of Emergency Management, Inje University, Gimhae 50834, Korea

**Keywords:** people with disabilities, disaster medicine, developed nations, developing nations, international organizations

## Abstract

Disability inclusion of children in disaster management means to identify and then eliminate the challenges faced by children with disabilities during disaster occurrence. The present research aimed to explore how the challenges of children with disabilities can be resolved in disaster management. Qualitative content analysis was used to compare individual-stakeholder-based disaster management with all-stakeholder disaster management considering three stakeholders: developed nations, developing nations, and international organizations. A key finding is that these stakeholders must shift from the individual-stakeholder-based approach to the all-stakeholders approach while enhancing disaster medicine, education, monitoring, and implementation stages. A comprehensive framework of disability inclusion is proposed to reflect effective disaster management for these children.

## 1. Introduction

Children with disabilities are abandoned or left behind before, during, or after a disaster, such as the coronavirus disease 2019 (COVID-19) pandemic [1]. Moreover, discrimination against or the maltreatment of such children seems to be the rule rather than the exception [2] (pp. 8–20). The occurrence of various disasters has further increased the extent of discrimination against children with disabilities, because disasters put children with disabilities at greater risk than children (or people) without disabilities. The issue of children with disabilities is not insignificant in the fields of disaster management, medical science (or disaster medicine), inclusive education, humanitarianism, and others.

Disaster includes both natural disasters (also known as natural hazards) and man-made emergencies [3]. Disaster management refers to government efforts to manage all kinds of hazards to ensure and protect the welfare of the people, with the support of various partners, including voluntary organizations, businesses, and local communities. A stakeholder is an individual (or independent party) who has a stake in or has been influenced by disaster management. A stakeholder influences and is influenced by disability inclusion and disaster management [4]. In particular, whereas an individual stakeholder represents a respective person or party, all stakeholders refers to various stakeholders altogether. Inclusion indicates that all stakeholders have to take appropriate actions to accommodate people with disabilities. Disability inclusion means to identify and then eliminate the challenges regarding people with disabilities [5]. Hence, children with disabilities should equally benefit from various disaster management activities, similarly to children without disabilities.

Besides, comprehensive disaster management consists of four factors—namely, the management of all kinds of hazards (including not only a natural disaster but also a human-made emergency), the participation of all stakeholders in disaster management (such as all kinds of professionals or the whole-community approach), the phases of the disaster management cycle (e.g., disaster prevention/mitigation, disaster preparedness, disaster response, and disaster recovery), and the management of all risks (including fatal human losses, economic impacts, psychological disorders, and other invisible impacts) [6] (pp. 1–12).

How children with disabilities are affected by a series of disaster events is variable depending on each individual case, with effects such as human loss, physical injuries, building collapse, mental disorder, unequal treatment, and others. Despite difficulties, those risks may be classified as three vulnerabilities: physical vulnerability, psychological vulnerability, and educational vulnerability [7,8]. Physical vulnerability includes not only human loss and but also physical injuries, while psychological vulnerability refers to the invisible psychological impact on children with disabilities in the short term. Educational vulnerability means that children with disabilities are particularly vulnerable to negative education, being followed by the occurrence of disasters.

Table 1 shows important numbers on the issue of children with disabilities with the support of three authors: the United Nations Office for Disaster Risk Reduction (UNISDR) (2014), Cornell University (2018), and Margaret A. Turk (2020). In summary, about 15% (93–150 million children) of all children in the world are those with disabilities, and the majority of nations have failed to include these children’s needs into an appropriate and significant part of the overall discussion on disaster management. In short, about 86% of children with disabilities have failed to participate in national disaster management and thus their fatality rates during disasters are higher by 4.3% than those of children without disabilities.

When clear and accurate information in accessible language is given to any child and appropriate training and exercise are repeatedly provided to them, they will be positively motivated to participate in disaster management at home, in their school, in the community, or in the nation [12]. Thus, children with disabilities should not be considered as liabilities (e.g., disabilities having a negative stigma in various regions, disabilities as disadvantages among thoughtless pupils, and others) in disaster management. Rather, including them in disaster management strategies such as emergency plans, emergency warning, etc., if the rest of nation allows, may help reduce the impacts of disasters.

The full inclusion of children with disabilities in disaster management strategies will benefit not only children with disabilities but also children without disabilities, adults with disabilities, and adults without disabilities [13,14]. From the perspective of disaster management, which broadly encompasses methods to deal with all types of hazards or related processes, such inclusion will benefit the entire population. Hence, a research question in the present study is “what are the most effective ways to enhance disability inclusion for children with disabilities in the field of emergency management?”. It is necessary for this study to follow scientific research steps in order to appropriately answer the above question. In general, scientific steps include reviewing previous literature, establishing groups of topics, identifying the gap in the literature, and trying to fill this gap.

This research aimed to investigate how children with disabilities can be included in the field of disaster management, with the ultimate goal of decreasing not only the human losses and economic damage caused by disasters but also the psychological impacts of disasters in various regions. Two styles of disaster management were compared—namely, individual-stakeholder-based and all stakeholders-based, considering three stakeholders: developed nations, developing nations, and international organizations. The three stakeholders may include all the important players in the field one way or another.

## 2. Methodology

One of the biggest challenges in the issue of disability inclusion in disaster management is the scarcity of empirical data [15] (pp. 167–212), partly because disabilities cannot be easily quantified. Moreover, some families and communities consider disabilities as a stigma and thus conceal them from the public. Some developed countries and many developing countries have not successfully translated even the available data into usable information due to conceptual ambiguity. The above fact was a motivation for this research to figure out an appropriate methodology.

This research applied qualitative content analysis as its key methodology. The aim of the qualitative content analysis in this study was to provide basic understanding and knowledge regarding the issue of disability inclusion in the field of disaster management. In other words, the qualitative content analysis was based on concepts, assumptions, themes, interpretations, and even individuals’ thoughts, rather than previous empirical data in order to describe the reality of disability inclusion in a subjective but scientific way. The following steps were observed: the formulation of a research question (what are the most effective ways to enhance disability inclusion for children with disabilities in the field of emergency management?), the identification of appropriate text materials (e.g., the title, subheadings, or organization of a paper help to group many English texts), the definition of the units of the analytical category (e.g., disabilities, disaster, disaster medicine, and disability inclusion), the coding of text materials (organizing or labeling texts according to different themes, such as individual-stakeholder-based disaster management versus all-stakeholder disaster management), the interpretation of text materials (interpreting and understanding the meanings of texts with the goal of disability inclusion), and the recording of the findings [16]. The qualitative content analysis thus involved the systematic interpretation or analysis of written texts.

Three internationally recognized search engines were used to obtain the qualitative texts—Google.com, EBSCOhost, and ScienceDirect—as shown in Table 2. Google.com uploaded many government documents on the research topic, whereas EBSCOhost provided a number of research articles. In doing so, the main documents for this study included both research articles and official documents, while minor documents included news articles and websites. ScienceDirect did not have many appropriate texts uploaded on the topic, but provided supplementary articles, such as health articles for people with disabilities. Several keywords were used to identify those main documents, including “barriers and alternative in disabilities and disaster”, “statistics on disabilities”, “disaster medicine”, “disaster preparedness and people with disabilities”, “children with disabilities and disaster”, and “how to improve disability inclusion”. The number of finally included data points was 40, to include 17 articles, 11 books, and 12 websites.

**Figure 1:**
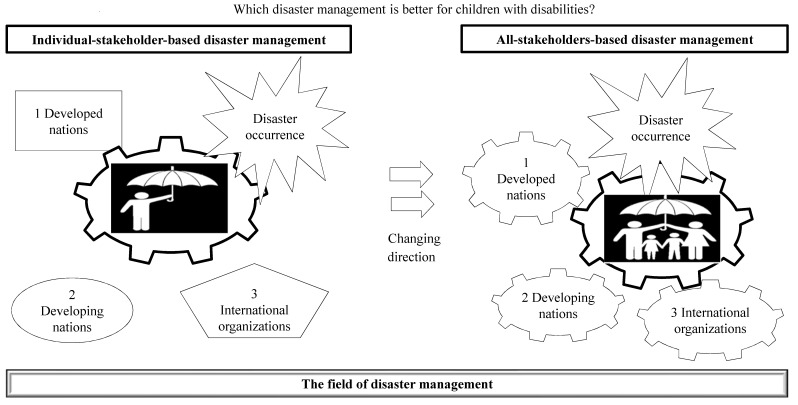
Analytical framework.

As shown in Figure 1, the present work investigates which type of disaster management—individual-stakeholder-based or all-stakeholder approach—has to be pursued. In the individual-stakeholder-based approach, each stakeholder carries out their own task, without coordinating their efforts with those of other stakeholders (similar to the function of three different diagrams around a toothed wheel). In the all-stakeholders approach, every stakeholder deals with disability inclusion in coordination with other stakeholders (similar to the function of three-toothed wheels around a toothed wheel). On the one hand, when the individual stakeholder includes a party which has been considered alone, they will not have a driving force in disability inclusion in the field of disaster management. On the other hand, because all stakeholders form a group or collection of individuals, they will have an impetus to deal with the topic.

Sub-variables were identified for the purpose of comparing and contrasting the two management styles. Three variables were selected—namely, developed nations, developing nations, and international organizations—with each of these stakeholders having their own justification for inclusion. Developed and developing nations were considered as major stakeholders in disability inclusion because all the nations of the world fall into either category. In general, developed nations have highly progressed their economies to include technological infrastructure, whereas developing nations have not done so yet. Accordingly, developing nations have a lower human development index and industrialization rate than developed nations. International organizations are included due to their involvement in disability inclusion between/among various nations. International organizations have been established which abide by international laws and treaties for the goal of cooperation in the field. They represent not only the UN but also other international institutions. The above three variables are large entities, but include small entities such as schools, parents, and others, when appropriate. Hence, these three stakeholders all play important roles in the field of disaster management.

## 3. Results

### 3.1. Individual-Stakeholder-Based Disaster Management

When addressing that the below three stakeholders have generally acted in the field independently, the approach is coded as individual-stakeholder-based disaster management.

#### 3.1.1. Developed Nations

The U.S. government, among many developed nations, has tried to address disability inclusion in disaster management; however, its efforts are far from complete yet, with measures such as rapid COVID-19 tests, appropriate vaccination for children with disabilities, and other disaster medicines. Additionally, disability inclusion has been a matter of importance during earthquakes, building fires, and other accidents. One such effort is involuntary or forced institutionalization, in which people with disabilities are kept in institutions without the provision of accessible shelters in integrated settings. Many children with disabilities have been unwillingly institutionalized during or after the occurrence of a disaster [17] (pp. 31–36). This removes children’s freedom in times of disaster when one or two stakeholders related to the institution make a related decision.

In 2017, residents in Gangseo-gu in Seoul, Korea, clashed on the issue of setting up a special school for children with disabilities in the region [18]. The majority of residents did not want to establish such a school, mainly because they believed it would decrease the price of local land. The other residents advocated the creation of a special school because of the reality of bullying at school, other discriminatory acts against disabilities, and the lack of emergency preparedness for multiple natural disasters. In particular, the parents of children with disabilities were among those who demanded a special school for their children.

#### 3.1.2. Developing Nations

Developing nations have struggled intensely with securing the rights and opportunities of children with disabilities. As poverty is intrinsically linked with disabilities, children are disproportionately disabled in poor regions (about 80% of children with disabilities live in developing nations) [19]. Many of these poor children live in rural areas, in particular without the supply of appropriate disaster medicine or medical treatment of disaster survivors. Disability inclusion has kept a similar pace with the issue of economic poverty in many senses. Without fundamentally addressing the matter of poverty as a core issue, disability inclusion may not be easily implemented, according to the UN Convention on the Rights of Persons with Disabilities (UNCRPD) [20] (pp. 29–40). However, the majority of developing nations have treated the issue of disabilities as a problem for individuals under the influence of a poor economy.

Disaster management education has been carried out in schools, in general. Additionally, those children with serious health issues (or emergency issues) are chronically absent from schools. Based on the above facts, a large proportion of children with disabilities have shown a low rate of school attendance in developing nations. For example, the percentage of children with disabilities who dropped out of school was roughly 23% in Uganda in 2011 compared with about 11% of children without disabilities. In Cambodia, almost 57% of children with disabilities were not in primary schools in 2014, compared with only 7% of children without disabilities [21] (pp. 6–9). These data indicate that governments, educators, or local communities have not systematically cooperated with children with disabilities or their families to decrease the school dropout rate.

#### 3.1.3. International Organizations

Several sub-institutions under the UN system deal with the issue of either disaster management or people with disabilities (e.g., the UN Office for Disaster Risk Reduction (UNDRR) for disaster management, the UN Office for the Coordination of Humanitarian Affairs (OCHA) for people with disabilities, the UN Children’s Fund (UNICEF) for all children, and others). Additionally, Article 11 of the UNCRPD addresses the issue of disability inclusion [22]. Many UN sub-institutions are oriented towards international connections, but their activities are not completely oriented for all stakeholders [23]. Instead, some activities have been held for individual stakeholders only once in a while due to limited resources. Similarly, those UN sub-institutions have not fully implemented their monitoring system for various regions.

A number of international nongovernmental organizations (NGOs) work toward disability inclusion in disaster management, as well as addressing the needs of children with disabilities. Many NGOs focus on their own areas of specialization (e.g., the Red Cross on sanitization and Handicap International on humanity and inclusion). However, although a few international NGOs recently joined the International Disability Alliance, the creation of partnerships among NGOs has generally been far from satisfactory. Due to inadequate staffing and funds, many NGOs struggle with integrating the issue of disability inclusion with other concerns in terms of related education and monitoring.

### 3.2. All-Stakeholders-Based Disaster Management

When thinking that the below three stakeholders need to systematically coordinate their efforts in the field, the approach is coded as all-stakeholders-based disaster management.

#### 3.2.1. Developed Nations

The U.S. Federal Emergency Management Agency (FEMA) and Department of Education (DoED) have provided guidelines on the principle of all-stakeholder disability inclusion [24]. Schools and local governments have also tried to meaningfully apply the principle in the field. Nonetheless, the establishment of a fair process, including involuntary institutionalization, has not been adequately addressed. Thus, not only governments but also schools need to further elaborate on the issue of all-stakeholder disaster management by enhancing the value proposition for each stakeholder, as well as improving COVID-19 vaccination and disaster medicine.

The case of Gangseo-gu in Korea showed few aspects of disability inclusion [25], because the clash between the two groups involved centered on the issue of whether or not a special school should be constructed for children with disabilities. In doing so, the parents of children with disabilities worried about school bullying and the lack of emergency preparedness, and hence demanded a separate school for their children, but also without considering disability inclusion. In some senses, most of those parents in Geanseo-gu did not even know the meaning of disability inclusion in 2017.

For the implementation of all-stakeholder disaster management, the field needs to meaningfully encourage children with disabilities to participate in decision-making. For example, Harry Shier (2001) proposed five steps of related participation: “(1) children are listened to, (2) children are supported in expressing their views, (3) children’s views are taken into account, (4) children are involved in decision-making processes, and (5) children share power and responsibility for decision-making” [26] (pp. 110–111). At each step, three kinds of commitment were noted to include not only openings and opportunities but also obligations.

#### 3.2.2. Developing Nations

Many developing nations have classified the issue of disabilities as an individual matter, mainly because they do not have the economic resources to deal with it [27] (pp. 210–214). In short, the hungry belly has no ears. However, as long as the issue of disabilities is viewed as a matter of poor economy, developing nations will never be able to adequately address disabilities or disability inclusion, as in the case of Brazil during the COVID-19 outbreak. Despite their poor economy, developing nations should regard the problem of children with disabilities as a societal issue so as to facilitate the involvement of various stakeholders regardless of economic status. A good example is the support for medical treatment during an emergency.

Fundamentally, developing nations need to pay more attention to taking care of children with disabilities in terms of disaster management than those without disabilities, when reflecting that the former possesses difficulties in moving and communicating [28]. For examples, families must prepare emergency kits, which include medicines, identification cards, water, and others, to keep their children calm. Healthcare professionals may help children with disabilities, while opening information channels with their families. Similarly, other stakeholders must try to let children with disabilities be informed of what is going on.

It is necessary for developing nations to reach out to children with disabilities in order to decrease their school dropout rate. Without children’s school attendance, disaster management for them would not be efficiently formed. Thus, the various stakeholders (or at least three people in each region) should listen to what children with disabilities want in a school regarding disability inclusion [29] (pp. 8–9). In other words, the field of disaster management must let the voice of children with disabilities be heard by multiple stakeholders through school programs, through equal emphasis on theories and practices.

#### 3.2.3. International Organizations

As a leading institution in the international community, the UN should place greater emphasis on the importance of including children with disabilities in disaster management. In other words, the UN should form a strong linkage between children with disabilities and disaster management. At present, the subject of disability inclusion is limited in scope. In particular, the UN has not focused on advocacy for children with disabilities as well as disaster management for children with disabilities. Without a UN vision on disability inclusion, the international community will not seriously address the issue [30]. Similarly, the multiple UN sub-institutions need to equally and substantially implement disability inclusion for children with disabilities.

To elaborate, it is necessary for the UN to rely on multiple methods while providing a UN vision and monitoring related sub-strategies. Among them, the issue of transformational leadership has been much supported regarding disability inclusion in the field of disaster management [31]. The leaders of the UN and its sub-institutions must work with diverse teams and then form a future-oriented vision for the subject. In doing so, the leaders need to figure out a UN vision by fully utilizing not only the rationale of scientific cause and effect, but also the data from multidisciplinary studies. Otherwise, the UN vision can not be carried out or will fail to be monitored in a substantial manner.

A greater awareness of disability inclusion is needed in order to expand the building of partnerships among international NGOs. After each international NGO defines its role and responsibility in the field, overlapping activities should be eliminated, or at least monitored, such as periodic tracking under a huge network. Transparency and balance will be a shortcut to achieving efficiency in partnership building [32] (pp. 1–4). These international NGOs could expand their partnerships with multiple UN sub-institutions as well as elementary schools and local communities.

### 3.3. Findings

To date, two distinctive types of disaster management have been rigorously compared—individual-stakeholder-based disaster management and all-stakeholder disaster management—in terms of the same three variables: developed nations, developing nations, and international organizations. The key finding is that all three stakeholders should shift from their current individual-stakeholder-based disaster management to an all-stakeholders approach.

In order to develop disaster resilience, the inclusion of not only the general public but also foreign-born employees, visitors, senior citizens, children, and people with disabilities is important. Developed nations have to review the whole process of disability inclusion, while developing nations should consider children with disabilities, disaster management, and school attendance as a societal issue. International organizations should further facilitate disability inclusion. Thus, adopting all-stakeholders disaster management would help to achieve a resilient community.

## 4. Discussion

Multiple challenges have, to date, prevented the field of disaster management from achieving its ultimate goal of reducing not only the physical impact of disasters, but also their social impact, which disability inclusion may help to accomplish [33,34]. The fact that all stakeholders play specific roles in including children with disabilities in the field is very helpful for not only those children but also other stakeholders. In particular, various barriers (e.g., physical barriers, communication barriers, attitudinal barriers, etc.), which children with disabilities have often faced, will decrease before, during, and after the occurrence of disasters. As inclusion will enable us to reduce the human loss and psychological impact caused by disasters, it will be a cornerstone for the whole field.

The qualitative data clearly indicate a need to shift from individual-stakeholder-based to all-stakeholders disaster management to include children with disabilities. (Manually coding text materials has been utilized in this paper. While relying on deductive coding, the authors repeatedly read text materials. After that, they categorized two codes: individual-stakeholder disaster management (five text data) vs. all-stakeholders disaster management (eight text data).) The present research thus contributes to the expansion of the existing literature, given that few researchers have attempted to provide a framework for the inclusion of children with disabilities in disaster management, and no major theories on the subject have thus far been developed. Moreover, the comparison of two styles of disaster management further clarifies the relationship between children with disabilities and disaster management.

The issue of disaster medicine as an independent specialty of medicine has been extremely significant for children with disabilities, regardless of national boundary. Disaster medicine includes not only emergency medical rescue but also emergency relief activities, which are basically different from the purpose of emergency services in hospitals [35]. Likewise, the aim of disaster medicine is to address many critical situations, such as critical human losses, multiple injuries, temporary medical facilities, rough working conditions, and other nonmedical features, while responding to those children’s special needs.

The transition is directly related to education because it is basically oriented toward children or young students with disabilities. By initiating inclusive education in which different students learn in the same classroom, and then educating them on the issue of disability inclusion in disaster management, educators could contribute extensively to the all-stakeholders approach. At the same time, educators may teach self-care and advocacy for child education. For adult education, educators need to convey mindset shifts about the rights of disabled children, how to support related families, inclusive education, and others. They could efficiently apply not only formal education tools, such as classroom learning and e-learning, but also informal education tools, such as holiday clubs and school trips.

The transition would require a series of disaster management training courses and exercises [36,37]. Planned training and exercises are implemented among children with disabilities to achieve the goal of disability inclusion. Among diverse programs, trainers who deal with children with disabilities will train adults in how to assist these children during a disaster. Through evaluation and feedback, concrete factors could then be identified to assist children with different disabilities [38].

To achieve a successful transition, the field should regularly monitor the process of disability inclusion by applying tools such as ratings and documentation [39] (pp. 88–95). The monitoring may be carried out internally or externally to thoroughly check the complicated process. Ratings provide relevant numbers for individuals and organizations; however, the decision made about these ratings should be based on a consensus. Documentation, through the use of various tables, plays a role in facilitating the understanding of the issue among stakeholders.

With all of the above implications in mind, the field may utilize implementation stages for a successful transition. In general, there are four implementation stages, consisting of an exploration stage, an installation stage, an initial implementation stage, and a full implementation stage [40]. To elaborate, the field must define the needs of disability inclusion in disaster management, and at the same time assess related readiness. After that, the field will build appropriate infrastructure and capacity for the transition. The field must start initial practices and programs, and then fully utilize them in order to achieve the original goal.

## 5. Conclusions

The present research aimed to investigate how to effectively include children with disabilities in disaster management to achieve the ultimate goal of decreasing the human losses, economic damage, and psychological impacts of disasters in various regions. Two disaster management styles—namely, the individual-stakeholder-based and the all-stakeholder approaches—were compared by considering three stakeholders: developed nations, developing nations, and international organizations. By presenting the challenges and proposing alternatives, the research achieved its objective.

A key finding is that the field of disaster management needs to shift from its current individual-stakeholder-based approach to an all-stakeholder approach to disability inclusion, while addressing disaster medicine, children’s rights to education, health, and care, as well as service, economy, and policy. To achieve this, not just one or two stakeholders but all three stakeholders must carry out their identified roles and responsibilities.

Disabilities are not immediately obvious, but researchers continue to analyze related matter these days, particularly in the 21st century. Nonetheless, the results of some studies on disabilities have not been so obvious. To the token, this study proved the importance of disability inclusion in the category of children with disabilities in disaster management as an advantage while considering the many aspects of disabled children.

Various academic fields tackle the issues of either children with disabilities or disability inclusion, such as education, humanitarianism, disaster medicine, and disaster management. Further research could apply the theme of disability inclusion proposed in this work to these other fields to increase the uptake of all-stakeholder disaster management in homes, cities, nations, and the international community, and ultimately accomplish the goal of decreasing not only the physical impacts of disasters but also their social impacts on children with disabilities.

## Figures and Tables

**Table 1:** Supplementary information on children with disabilities [9,10,11].

Units	Empirical Data	Remarks
Percentage of people with disabilities in the world	About 15% of the world population	About 2–4% of people with disabilities have experienced significant difficulties
Number of children with disabilities	About 93–150 million children	About 14% of all children have disabilities
Percentage of children with disabilities who have not participated in disaster management in the community	About 86% of children with disabilities	Based on respondents from 137 countries
Comparison of fatality rates during disasters between children with disabilities and children without disabilities	4.3% higher in children with disabilities than in children without disabilities	Good examples are the occurrence of an earthquake and tsunami in Fukushima, Japan, in 2011, and the outbreak of COVID-19 in the United States in 2020. (The latter is for children with intellectual and developmental disability, not all disabilities.)

**Table 2:** Qualitative data information under the integrative review.

Major Items	Research Period
	9 October 2020–2 July 2021
➀ Number of initially identified data points	159
➁ Number of removed data points	119
➂ Number of finally included data points	40
➃ Number of coding text data points	5 vs. 8 (individual-stakeholder-based disaster management vs. all stakeholders-based disaster management)
➄ Criteria for data inclusion	The issues of children with disabilities, disaster management, and others
➅ Criteria for data removal	Those issues which limit the interpretability of research goals and results
➆ Utilized databases	Google.com, EBSCOhost, and ScienceDirect

## Data Availability

No new data were created or analyzed in this study. Data sharing is not applicable to this article.

## Abbreviations

The following is an abbreviation list for this manuscript:

CDC: Centers for Disease Control and Prevention; DoED: Department of Education; FEMA: Federal Emergency Management Agency; GFDRR: Global Facility for Disaster Reduction and Recovery; IFRC: International Federation of Red Cross and Red Crescent Societies; IIRR: International Institute of Rural Reconstruction; NCD: National Council on Disability; NGOs: nongovernmental organizations; NIRM: National Implementation Research Network; OCHA: United Nations Office for the Coordination of Humanitarian Affairs; UN: United Nations; UNCRPD: United Nations Convention on the Rights of Persons with Disabilities; UNDRR: United Nations Office for Disaster Risk Reduction; UNESCO: United Nations Educational, Scientific and Cultural Organization; UNICEF: United Nations Children’s Fund; UNISDR: United Nations Office for Disaster Risk Reduction; WHO: World Health Organization.
